# Nutritional intake and metabolic parameters of Japanese university students with and without obesity: Sex-specific differences

**DOI:** 10.1371/journal.pone.0285088

**Published:** 2023-05-03

**Authors:** Mausam Mehta, Ricardo Izurieta, Akihiro Nishio, Ryo Horita, Mayumi Yamamoto

**Affiliations:** 1 Morsani College of Medicine, University of South Florida, Tampa, Florida, United States of America; 2 Department of Global Health, College of Public Health, University of South Florida, Tampa, Florida, United States of America; 3 Health Administration Center, Gifu University, Gifu, Japan; 4 Department of Psychiatry, Gifu University Hospital, Gifu, Japan; 5 Department of Endocrinology and Metabolism, Gifu University Hospital, Gifu, Japan; 6 United Graduate School of Drug Discovery and Medical information Sciences, Gifu University, Gifu, Japan; Auburn University, UNITED STATES

## Abstract

**Objective:**

To establish effective methods of obesity prevention among young adults, we analyzed the relationship between obesity-related food intake and metabolic factors in Japanese university students.

**Methods:**

We performed a cross-sectional analysis of nutrient intake information and metabolic parameters among 1,206 Gifu University students categorized by body mass index.

**Results:**

The overweight/obesity rate was significantly higher in males. Additionally, among males, the intake of protein, potassium, magnesium, phosphorus, iron, zinc, all lipids/fats, and all metabolic parameters including blood sugar, hemoglobin A1c, uric acid, alanine aminotransferase, aspartate aminotransferase, low-density lipoprotein, high-density lipoprotein, triglycerides, and blood pressure significantly differed between the obese and non-obese groups. However, the same comparison among females revealed no significant differences in nutrient intake and significant differences in only half of the parameters. Among males, energy intake from protein and fat was significantly higher in the obese group, while the percentage of total energy intake from carbohydrates and fat was lower and higher, respectively, among females in the obese group.

**Conclusions:**

Overeating of protein and fat in males and unbalanced nutrition in females are sex-specific characteristics of Japanese university students with obesity, and metabolic abnormalities in students with obesity are more remarkable in males than in females.

## Introduction

Overweight status, obesity, and a rapid increase in the prevalence and disease burden of an elevated body mass index (BMI) are important clinical and public health concerns worldwide [1,2]. A national survey in the U.S. demonstrated that severe obesity was associated with an increased prevalence of cardiometabolic risk factors in boys and young men [3]. Additionally, in Japan, a population-based cohort study showed an increasing prevalence of obesity in younger men [4].

In the face of the global obesity epidemic that is causing population-wide adverse health effects, morbidity, and mortality, the need for the prevention of and interventions for obesity in young adults is urgent. However, evidence-based guidelines for interventional programs targeted at this generation have not been established due to limited access to data for this group. Although a few surveys have demonstrated the weight status [5–8] and dietary intake [6-‍9] of young adults in the U.S., there are few reports relating obesity and dietary intake among young Japanese adults.

To establish effective methods of obesity prevention among young Japanese adults, the relationship between nutritional intake and various metabolic factors of Japanese university students falling within each BMI category was analyzed. We hypothesized that there are significant differences in the nutritional intake and health outcomes in Japanese university students with different BMIs (lean, normal weight, and obesity).

## Methods

### Ethical considerations

All study procedures complied with the ethical requirements of the national and institutional committees that oversee human studies, as well as with the 1964 Declaration of Helsinki and its later revisions. The study design was reviewed and approved by the Ethical Review Committee of the Graduate School of Medicine, Gifu University, Japan (no. 26–331). All participants provided written consent.

### Participants and study procedures

To elucidate the feature of nutritional intake and real-time health data among young Japanese adults in different BMI categories, a cross-sectional analysis was conducted on Gifu University’s incoming class of 2017. Nutritional intake information was obtained and quantified using the self-administered brief-type diet history questionnaire (BDHQ) that has been adjusted and validated for the Japanese population [10]. In addition, participants’ backgrounds (self-reported sex, age, smoking and drinking status, and present illness) and metabolic parameter data were collected during the annual health checkup, which is mandated by the Japanese School Health and Safety Act for all Japanese students. To collect information on smoking and drinking status, all students were asked the following questions: “Did you engage in any smoking, including electronic cigarettes, in the previous month?” (choices: 1 = No, 2 = Yes) and “How many days per week do you consume alcohol on average?” (choices: 1 = 0 days, 2 = 1–2 day(s), 3 = 3–4 days, 4 = 5–6 days, 5 = everyday). We categorized participants who selected choice 1 as non-smokers or non-drinkers, and those who selected other choices as smokers or habitual drinkers.

The following inclusion criteria were used to select the charts that would be included in the analysis: an incoming Gifu University student of 2017, aged 18–30 years, self-identification as Japanese, finished all examination items including the biological sex question and the BDHQ, as well as having had a complete annual health checkup. The major exclusion criteria were as follows: international students, current eating disorders, pregnancy or lactation, on birth control, or current major medical conditions. Among a total of 1,277 charts (checkup rate: 99%), 1,206 (746 males and 460 females, aged 20.36 ± 2.17 years) met the inclusion criteria and were included in the analysis. Nutrition and real-time health data from these individuals were categorized according to sex (male and female) and BMI (lean, normal weight, and obesity). BMI categories were defined according to the criteria from the Japan Society for the Study of Obesity [11] as follows: lean, BMI<18.5 kg/m^2^; normal weight, BMI of 18.5 kg/m^2^–24.9 kg/m^2^; and obese, BMI≥25.0 kg/m^2^.

Nutritional intake variables that were analyzed included the following 21 parameters: total energy (kJ/day), water (g/day), protein (g/day), carbohydrates (g/day), sucrose (g/day), total dietary fiber (g/day), and salt (all measured in mg/day: sodium, potassium [K], calcium [Ca], magnesium (Mg), phosphorus [P], iron [Fe], zinc [Zn], copper [Cu], manganese [Mn]), total fat (g/day), saturated fatty acids (g/day), mono-/polyunsaturated fatty acids (g/day), omega-3 (n-3)/omega-6 (n-6) fatty acids (g/day), and total cholesterol (g/day). Metabolic parameters that were analyzed included the following 10 parameters: blood sugar (BS), hemoglobin A1c (HbA1c) (%), uric acid (UA), low-density lipoprotein (LDL), high-density lipoprotein (HDL) cholesterol, triglycerides (TG) (all measured in mg/dL), systolic and diastolic blood pressure (BP) (mmHg), aspartate aminotransferase (AST), and alanine aminotransferase (ALT) (both measured in IU/L).

To compare nutritional intake and metabolic parameters between each different BMI category among the students, the data of all nutritional and metabolic parameters were categorized according to sex and BMI and subsequently analyzed.

### Statistical analyses

To compare the variables between the groups, we performed one-way ANOVA with post-hoc Tukey honestly significant difference analysis using SPSS version 24 software (IBM Corporation, Armonk, New York). To show the sex differences, linear regression models were used to examine nutrient intake and metabolic parameters. The models used age, BMI, smoking status, and drinking habits as the independent variables and were performed using StatFlex version 7 software (Artech Co Ltd., Osaka, Japan). Data were considered statistically significant at p<0.05.

## Results

Information on the demographic characteristics of the participants is shown in Table 1. Of the 746 males (age: 20.66 ± 2.23 years), 25 (3.4%) were actively smoking, 128 (17.2%) were drinking alcohol habitually, and 104 (14.0%), 543 (72.8%), and 979 (13.3%) were classified as lean, normal weight, and obese, respectively. Furthermore, of the 460 females (age: 19.88 ± 1.99 years), 2 (0.4%) were actively smoking, 31 (6.7%) were drinking alcohol habitually, and 81 (17.6%), 362 (78.7%), and 17 (3.7%) were classified as lean, normal weight, and obese, respectively. There was a significant difference between males and females in the proportion of students who smoked, drank alcohol, and were obese (Table 1).

**Table 1:** Characteristics of the study participants.

	Participants (n = 1206)			
	Male		Female	
	n (%)	Age (years)	n (%)	Age (years)
Total	746 (100.0)	20.66 ± 2.23	460 (100.0)	19.88 ± 1.99
Current smoking	25 (3.4)*	23.24 ± 1.09	2 (0.4)	23.00 ± 1.41
Habitual drinking	128 (17.2)*	23.13 ± 0.96	31 (6.7)	23.19 ± 1.11
Category (BMI; kg/m^2^)				
Lean (<18.5)	104 (14.0)	20.17 ± 2.16	81 (17.6)	20.11 ± 2.15
Normal (18.5–24.9)	543 (72.8)	20.72 ± 2.23	362 (78.7)	19.79 ± 1.92
Obese (≥25.0)	99 (13.3)*	20.78 ± 2.28	17 (3.7)	20.53 ± 2.43

Data are presented as means ± standard deviation.

*Analyzed by one-way ANOVA with post-hoc Tukey honestly significant difference analysis, statistically significant difference to females (p<0.01); BMI, body mass index.

Among males, there were significant differences between the groups with and without obesity regarding the following nutritional intake parameters: protein, K, Mg, P, Fe, Zn, total fat, saturated fat, mono-/poly-unsaturated fatty acids, n-3/n-6 fatty acids, and total cholesterol. The group with obesity had a significantly higher intake of these nutrients than the group without obesity. In contrast, among women, there were no significant differences between the BMI categories regarding any of the nutritional intake values. The linear regression models included age, smoking status, alcohol drinking, BMI, and sex as independent variables, and revealed sex differences associated with nutrition intake according to BMI. Significant interactions (p<0.05) between sex and BMI were demonstrated in total energy, water, protein, K, Ca, Mg, P, Fe, Zn, Cu, total fat, saturated fatty acid, mono-/poly-unsaturated fatty acid, n-3/n-6 fatty acid, and total cholesterol (Table 2).

**Table 2:** Nutrition intake of the participants according to BMI.

		BMI (kg/m^2^)									
		Lean (<18.5)			Normal (18.5–24.9)			Obese (≥25.0)			
Males											
	**Total energy (kJ/day)**	**8,273.8**	**±**	**2,732.3**	**8,901.4**	**±**	**3,079.3**	**8,998.7**	**±**	**2,712.3**	
	**Water (g/day)**	**1,702.7**	**±**	**666.8**	**1,822.5**	**±**	**701.1**	**1,893.0**	**±**	**745.2**	
	**Protein (g/day)**	**69.5**	**±**	**26.7**	**76.9**	**±**	**30.8**	**81.1**	**±**	**30.8**	*
	Carbohydrate (g/day)	287.1	±	109.9	302.1	±	117.2	294.7	±	97.4	
	Sucrose (g/day)	11.6	±	8.6	11.4	±	9.1	9.8	±	5.7	
	Total dietary fiber (g/day)	10.7	±	4.3	11.6	±	5.3	11.7	±	5.1	
	Salt (g/day)	11.0	±	3.6	11.5	±	3.8	11.6	±	3.6	
	Na (mg/day)	4,332.6	±	1,437.0	4,542.8	±	1,520.7	4,581.4	±	1,409.3	
	**K (mg/day)**	**2,163.7**	**±**	**954.6**	**2,411.7**	**±**	**1,115.2**	**2,557.2**	**±**	**1,056.8**	*
	**Ca (mg/day)**	**468.6**	**±**	**237.0**	**525.0**	**±**	**283.4**	**547.0**	**±**	**277.2**	
	**Mg (mg/day)**	**218.3**	**±**	**87.1**	**242.0**	**±**	**102.2**	**254.9**	**±**	**101.1**	*
	**P (mg/day)**	**989.6**	**±**	**398.8**	**1,100.6**	**±**	**468.0**	**1,164.0**	**±**	**459.9**	*
	**Fe (mg/day)**	**7.3**	**±**	**2.8**	**8.0**	**±**	**3.5**	**8.4**	**±**	**3.7**	*
	**Zn (mg/day)**	**8.6**	**±**	**2.8**	**9.5**	**±**	**3.7**	**9.9**	**±**	**3.5**	*
	**Cu (mg/day)**	**1.2**	**±**	**0.4**	**1.3**	**±**	**0.5**	**1.3**	**±**	**0.5**	
	Mn (mg/day)	3.4	±	1.2	3.5	±	1.5	3.6	±	1.5	
	**Total fat (g/day)**	**56.6**	**±**	**21.6**	**61.7**	**±**	**24.9**	**65.4**	**±**	**25.2**	*
	**Saturated fatty acid (g/day)**	**15.2**	**±**	**6.4**	**16.9**	**±**	**7.7**	**17.7**	**±**	**7.4**	*
	**Mono-unsaturated fatty acid (g/day)**	**20.7**	**±**	**7.9**	**22.4**	**±**	**9.1**	**23.9**	**±**	**9.3**	*
	**Poly-unsaturated fatty acid (g/day)**	**13.6**	**±**	**5.1**	**14.7**	**±**	**5.7**	**15.6**	**±**	**6.2**	*
	**n-3 fatty acid (g/day)**	**2.5**	**±**	**1.2**	**2.7**	**±**	**1.3**	**3.0**	**±**	**1.3**	*
	**n-6 fatty acid (g/day)**	**11.1**	**±**	**3.9**	**12.0**	**±**	**4.6**	**12.6**	**±**	**4.9**	*
	**Total cholesterol (mg/day)**	**377.8**	**±**	**181.5**	**427.6**	**±**	**221.6**	**467.4**	**±**	**236.6**	*
Females											
	**Total energy (kJ/day)**	**6,909.0**	**±**	**1,858.4**	**6,813.1**	**±**	**2,075.5**	**7,209.1**	**±**	**2,225.7**	
	**Water (g/day)**	**1,467.6**	**±**	**429.7**	**1,467.4**	**±**	**521.3**	**1,502.0**	**±**	**467.2**	
	**Protein (g/day)**	**62.4**	**±**	**20.7**	**62.2**	**±**	**22.6**	**70.7**	**±**	**30.3**	
	Carbohydrate (g/day)	231.7	±	70.8	221.1	±	74.3	220.9	±	67.7	
	Sucrose (g/day)	12.5	±	9.4	11.4	±	8.0	13.6	±	8.7	
	Total dietary fiber (g/day)	10.6	±	3.9	10.6	±	4.5	10.7	±	4.7	
	Salt (g/day)	9.2	±	2.7	9.1	±	3.1	9.8	±	2.2	
	Na (mg/day)	3,654.8	±	1,074.1	3,602.0	±	1,218.5	3,878.2	±	862.7	
	**K (mg/day)**	**2,126.7**	**±**	**797.7**	**2,173.2**	**±**	**905.2**	**2,380.9**	**±**	**1,146.0**	
	**Ca (mg/day)**	**446.0**	**±**	**180.9**	**463.8**	**±**	**204.8**	**499.9**	**±**	**285.3**	
	**Mg (mg/day)**	**207.6**	**±**	**73.4**	**206.6**	**±**	**79.3**	**218.2**	**±**	**99.1**	
	**P (mg/day)**	**907.6**	**±**	**315.1**	**914.1**	**±**	**337.7**	**1,029.9**	**±**	**460.6**	
	**Fe (mg/day)**	**7.0**	**±**	**2.5**	**7.0**	**±**	**2.8**	**7.3**	**±**	**3.0**	
	**Zn (mg/day)**	**7.5**	**±**	**2.3**	**7.5**	**±**	**2.5**	**8.1**	**±**	**3.1**	
	**Cu (mg/day)**	**1.1**	**±**	**0.3**	**1.0**	**±**	**0.3**	**1.0**	**±**	**0.4**	
	Mn (mg/day)	3.0	±	1.0	2.9	±	1.1	2.8	±	1.0	
	**Total fat (g/day)**	**49.4**	**±**	**14.8**	**52.1**	**±**	**19.2**	**59.1**	**±**	**20.1**	
	**Saturated fatty acid (g/day)**	**13.5**	**±**	**4.5**	**14.4**	**±**	**5.6**	**16.6**	**±**	**6.7**	
	**Mono-unsaturated fatty acid (g/day)**	**17.8**	**±**	**5.3**	**18.9**	**±**	**7.3**	**21.6**	**±**	**7.2**	
	**Poly-unsaturated fatty acid (g/day)**	**11.6**	**±**	**3.7**	**12.2**	**±**	**4.6**	**13.3**	**±**	**3.8**	
	**n-3 fatty acid (g/day)**	**2.2**	**±**	**0.9**	**2.3**	**±**	**1.0**	**2.5**	**±**	**0.8**	
	**n-6 fatty acid (g/day)**	**9.4**	**±**	**2.8**	**9.9**	**±**	**3.7**	**10.8**	**±**	**3.1**	
	**Total cholesterol (mg/day)**	**379.5**	**±**	**161.2**	**382.5**	**±**	**168.5**	**435.7**	**±**	**210.0**	

Data are presented as means ± standard deviation.

*Statistically significant difference (p<0.05) analyzed by one-way ANOVA using the post-hoc Tukey honestly significant difference analysis between the three groups.

Sex differences associated with nutrition intake according to BMI were analyzed using linear regression models which included age, smoking status, alcohol drinking, BMI, and sex as independent variables.

Significant interactions between sex and BMI (p<0.05) were shown for total energy, water, protein, K, Ca, Mg, P, Fe, Zn, Cu, total fat, saturated fatty acid, mono-/poly-unsaturated fatty acid, n-3/n-6 fatty acid, and total cholesterol, which are highlighted in bold face type.

BMI, body mass index; Na, sodium; K, potassium; Ca, Calcium; Mg, magnesium; P, phosphorus Fe, Iron; Zn, Zinc; Cu, Copper; Mn, Manganese; n-3/n-6, omega-3/omega-6.

The percentage of energy intake (%) from carbohydrates, protein, and fat per the daily total energy intake are compiled in Table 3. Among the males, there were no significant differences in the percentage of energy intake (%) from carbohydrates, protein, and fat between the BMI categories. In contrast, among females, the percentage of energy intake (%) from carbohydrates was significantly lower and that of fat was significantly higher in the group with obesity than in the group without obesity (Table 3).

**Table 3:** Percentage of total daily energy intake of carbohydrate, protein, and fat according to BMI.

	BMI		
	Lean (<18.5)	Normal (18.5–24.9)	Obese (≥25.0)
Males			
Total energy	100.0 ± 33.0	100.0 ± 34.6	100.0 ± 30.1
**Carbohydrate**	**58.1 ± 22.2**	**56.8 ± 22.1**	**54.8 ± 18.1**
Protein	14.1 ± 5.4	14.5 ± 5.8	15.1 ± 5.7
**Fat**	**25.8 ± 9.8**	**26.1 ± 10.5**	**27.4 ± 10.6**
Females			
Total energy	100.0 ± 26.9	100.0 ± 30.5	100.0 ± 30.9
**Carbohydrate**	**56.1 ± 17.2**	**54.3 ± 18.3**	**51.3 ± 15.7** *
Protein	15.1 ± 5.0	15.3 ± 5.6	16.4 ± 7.0
**Fat**	**26.9 ± 8.0**	**28.8 ± 10.6**	**30.9 ± 10.5** *

Data are presented as means ± standard deviation.

*Statistically significant difference to non-obese groups (p<0.05).

Sex differences associated with percentages of nutrition intake according to BMI were analyzed using linear regression models which included age, smoking status, alcohol drinking, BMI, and sex as independent variables.

Significant interactions (p<0.05) between sex and BMI are shown in the percentages of total daily energy intake of carbohydrate and fat, which are highlighted by bold face type.

BMI, body mass index.

Among males, there were significant differences in obesity regarding all 10 metabolic parameters, including BS, HbA1c, UA, LDL and HDL cholesterol, TG, systolic/diastolic BP, AST, and ALT, between the lean and normal weight BMI categories and the obese group. Additionally, the group with obesity had significantly higher metabolic parameter values than the other groups, except for HDL cholesterol, where a significantly lower value was observed. In contrast, among the females, there were significant differences in only five metabolic parameters, including UA, LDL and HDL cholesterol, TG, and systolic BP, between the lean and normal weight BMI categories and the obese group (Table 4).

**Table 4:** Obesity-related metabolic parameters according to BMI.

	BMI (kg/m^2^)			
	Lean (<18.5)	Normal (18.5–24.9)	Obese (≥25.0)	p
Males				
BS (mg/dl)	89.7 ± 11.6	88.2 ± 13.3	92.5 ± 11.6	0.01*
HbA1c (%)	5.0 ± 0.2	5.0 ± 0.2	5.1 ± 0.2	<0.0001***
**UA (mg/dl)**	**5.4 ± 1.0**	**5.7 ± 1.0**	**6.6 ± 1.2**	**<0.0001** ***
ALT (IU/L)	16.1 ± 6.1	23.2 ± 23.6	51.7 ± 44.6	<0.0001***
AST (IU/L)	19.7 ± 5.8	21.4± 10.7	31.6 ± 21.0	<0.0001***
LDL cholesterol (mg/dl)	83.9 ± 18.8	93.9 ± 23.3	116.4 ± 32.2	<0.0001***
**HDL cholesterol (mg/dl)**	**60.6 ± 10.2**	**57.7 ± 11.6**	**50.4 ± 11.3**	**<0.0001** ***
TG (mg/dl)	73.8 ± 36.0	93.7± 56.5	143.9 ± 91.0	<0.0001***
**Systolic BP (mmHg)**	**120.3 ± 11.4**	**124.7 ± 10.1**	**131.4 ± 9.6**	**<0.0001** ***
**Diastolic BP (mmHg)**	**68.5 ± 7.8**	**68.9 ± 7.8**	**71.8 ± 8.1**	**0.002** **
Females				
BS (mg/dl)	87.7 ± 9.8	86.6 ± 10.6	89.3± 11.2	0.445
HbA1c (%)	5.1 ± 0.2	5.1 ± 0.2	5.0 ± 0.3	0.714
**UA (mg/dl)**	**4.0 ± 0.8**	**4.2 ± 0.8**	**5.0 ± 1.1**	**<0.0001** ***
ALT (IU/L)	13.8 ± 8.0	13.6± 7.4	18.4 ± 8.1	0.513
AST (IU/L)	19.1 ± 6.2	18.5 ± 4.5	19.0 ± 5.2	0.41
LDL cholesterol (mg/dl)	94.1 ± 22.2	95.1 ± 22.9	111.9 ± 33.8	0.012*
**HDL cholesterol (mg/dl)**	**72.0 ± 15.3**	**68.0 ± 13.8**	**60.4 ± 14.4**	**0.004** **
TG (mg/dl)	62.1 ± 38.8	68.2 ± 36.4	113.9 ± 89.0	<0.0001***
**Systolic BP (mmHg)**	**113.4 ± 11.8**	**114.8 ± 11.3**	**121.7 ± 12.4**	**0.025** *
**Diastolic BP (mmHg)**	**64.9 ± 8.4**	**65.6 ± 8.2**	**68.8 ± 9.0**	**0.196**

Data are presented as means ± standard deviation.

*p<0.05, **p<0.01, ***p<0.001: Statistically significant differences analyzed by one-way ANOVA with post-hoc Tukey honestly significant difference analysis between the three groups.

Sex differences associated with metabolic parameters according to BMI were analyzed using linear regression models which included age, smoking status, alcohol drinking, BMI, and sex as independent variables.

Significant interactions (p<0.05) between sex and BMI are shown in UA, HDL cholesterol, and systolic/diastolic BP, which are highlighted in bold face type.

BMI, body mass index; BS, blood sugar; HbA1c, hemoglobinA1c; UA, uric acid; AST, aspartate aminotransferase; ALT, alanine aminotransferase; BP, blood pressure; LDL, low-density lipoprotein; HDL, high-density lipoprotein; TG, triglycerides; Data are means ± standard deviation (SD).

Linear regression models that included sex, age, BMI, smoking status, and habitual alcohol drinking as independent variables were used to assess sex differences in the 10 metabolic parameters related to obesity, including BS, HbA1c, UA, LDL/HDL cholesterol, TG, systolic/diastolic BP, AST, and ALT. Sex was a significant explanatory variable for UA, HDL cholesterol, and systolic/diastolic BP, similar to BMI, which was an explanatory variable for LDL/HDL cholesterol and systolic/diastolic BP (Table 4).

## Discussion

To our knowledge, this study was the first to examine sex differences in the prevalence of obesity, nutritional intake features, and obesity-related metabolic parameters among Japanese university students. We found obesity to be significantly less prevalent among females than among males. Moreover, excessive energy intake in males and nutritional intake imbalance (insufficient carbohydrate and excessive fat intake) in females seemed to be the characteristics of obesity-related nutritional intake. We found that the metabolic parameters differed according to sex; interestingly, females with obesity had fewer abnormal metabolic parameters than males with obesity.

In this study, we found that only 3.7% of female Japanese university students were obese, which was a third of the percentage of males with obesity. Previously, a cross-sectional study in a large southeastern university in North Carolina reported that 48.1% of male and 28.9% of female enrolled college students had a BMI≥25.0 [5]. Although the rate of overweight/obesity (BMI≥25.0) in American students was significantly higher than that in Japanese students, the prevalence of obesity was significantly higher in male than in female American students, similar to our study trend in Japanese students [5–7].

Herein, we describe the detailed nutritional intake features of Japanese university students according to sex and BMI classification. Although several reports have demonstrated the dietary habits of American university students [6–9], there have been no detailed reports on nutrient intake. A few studies have examined the relationship between dietary habits, including eating out [12]; regular egg consumption at breakfast [13]; and meals combining a staple food, main dish, and side dish [14], and nutrient intake in Japanese university students. However, these three surveys did not analyze the BMI, and the former two surveys were conducted only in female students, while the latter contained data for both sexes but the data were not analyzed according to sex. Therefore, to our knowledge, this study is the first to demonstrate a significant increase in the intake of various nutrients among male university students with obesity, and a significantly imbalanced intake (low carbohydrate and high fat intake) among female university students with obesity in Japan.

Based on these study results, we suggest the necessity of sex-specific educational interventions for obesity prevention to maximize its effectiveness. More specifically, males should be educated on preventing overeating and females on improving nutritional balance. Although Li et al. [15] did not demonstrate nutrient intake, their study among Chinese university students showed that males needed to reduce the fat content of their diets and females needed to increase the consumption of fiber, fruit, and vegetables. Furthermore, Von Bothmer and Fridlund [16] showed a high level of obesity and disinterest in nutrition advice among male Swedish university students. These findings support our proposal.

Cultural sex-specific differences or biological sex differences in eating behavior and body weight control have been reported among other populations [15–23]; however, this was not among university students. Generally, females are more conscious of their health, making them more likely to accept dietary recommendations [17], choose healthier foods, and consume fruit as a snack [18]. Although sex-specific patterns of dietary intakes and eating behaviors have been explained by eating-related self-determination [19], chewing performance [20], and eating-related and psychosocial stress [21], sex-specific biological factors and sex-related social factors may interact reciprocally on the individual’s diet [22]. When designing nutritional education for university students, we must consider sex-specific differences in dietary behavior, style of nutrition, dietary profile, eating location, and sources of nutrition knowledge [23].

We also showed that all 10 metabolic parameters were significantly different between the group with obesity and the group without obesity among males, whereas only 5 metabolic parameters were significantly different between these groups in females. This difference could be explained by both sex-related social factors and sex-specific biological factors. In fact, our new findings in Japanese university students with obesity suggest that overeating among males and a nutrition imbalance among females are characteristic differences that could be explained by sex-related social factors. Additionally, we may consider that sex-related differences in dietary intake may induce sex-related differences in metabolic parameter values. Simultaneously, some sex-specific biological factors might affect the differences in metabolic parameters between males and females, for example, sex hormones in adiposity and inflammation in obesity [24], estrogen receptors as regulators of body weight and insulin sensitivity [25], higher leptin and adiponectin concentrations in females than in males [26], and gonadal hormones and sex chromosome complement that contribute to lipid metabolism [27]. However, we could not investigate sex-specific biological factors in participants in this study; therefore, further research is required.

Many researchers have warned about the dramatically increasing prevalence of overweight status and obesity [1,2], especially among children and young adults [3]. Previously, through a 10-year follow-up of a population-based cohort study, an increasing prevalence of obesity was observed among younger generations in Japanese males [4]. Health promotion focusing on weight management on campus is an urgent need for university students because it may be the last chance for students to have food and dietary education before employment. A previous study examined the association between weight management goals and physical activity or diet in U.S. high school students [28]; however, there are few reports providing information on weight and diet on university campuses. Therefore, this study could provide the crucial information—that is, appropriate energy intake for males and balanced nutrition for females—for weight control promotion programs conducted on university campus.

This study had one notable limitation. We could not elucidate the clear mechanism of sex-related differences in the metabolic parameter values because we did not measure suspected biological factors, including various hormones such as androgens, adiponectin, and leptin. Additional studies on these factors are needed in the future.

## Conclusion

In conclusion, overeating among males and unbalanced nutrition among females are sex-specific differences characteristic of Japanese university students with obesity. Additionally, the observed metabolic abnormalities were more remarkable in obese males than in obese females.
